# 血管正常化与肿瘤免疫治疗

**DOI:** 10.3779/j.issn.1009-3419.2014.03.16

**Published:** 2014-03-20

**Authors:** 俊莉 曾, 冬梅 袁, 红兵 刘, 勇 宋

**Affiliations:** 1 510515 广州，南方医科大学研究生院 Graduate School, Nanfang Medical University, 510515 Guangzhou, China; 2 210002 南京，南方医科大学研究生院，南京军区南京总院呼吸内科 Department of Respiratory Medicine, Nanjing General Hospital of Nanjing Command, Nanjing 210002, China

**Keywords:** 异常血管形成, 免疫抑制微环境, 血管正常化, 抗肿瘤免疫, Abnormal angiogenensis, Immunosuppressive microenvironment, Vascular normalization, Anticancer immunotherapy

## Abstract

免疫治疗是一种颇有前景的抗肿瘤策略。然而，肿瘤中的免疫抑制微环境阻碍了免疫治疗的发展。异常肿瘤血管造成的缺氧，使免疫细胞趋向免疫抑制。并且异常血管通过分泌生长因子及细胞因子，改变免疫细胞的增殖、分化及功能，最终形成免疫抑制的微环境。因此，有效的利用血管生成及肿瘤免疫之间的相互作用，适当的抑制血管形成，促进肿瘤血管正常化，可以改变肿瘤的免疫抑制微环境，成为改善免疫治疗的新策略。现就血管正常化与肿瘤免疫的关系，及二者的联合治疗进行综述。。

## Abstract (en)

Immunotherapies, as a promising anticancer therapy stratrgy, has been paid more and more attentions. However, the abnormal tumor vasculature creates a hypoxic microenvironment that make immune cells toward immune suppression. The immunosuppressive microenvironment seems to impede the development of immunotherapies. Hence, normalization tumor vascular by anti-angiogenesis properly could improve the immunosuppressive miroenvironment. Consequently, the efficacy of the immunotherapies was enhanced. Here, we discuss the effects of vascular normalizing on tumor immunity and propose a potentially strategy to re-engineer the tumor-immune microenvironment and improve cancer immunotherapy.

血管生成是肿瘤生长的一个重要过程，在正常组织中，促血管生成及抗血管生成处于平衡状态，但是在肿瘤中，这种平衡被打破。肿瘤血管结构紊乱：微血管密度增加；血管形态迂曲，膨胀，呈囊状；周细胞（perivascular cell, PVC）形态异常，功能不足，连接松散甚至缺如；基底膜不完整，且厚薄不均^[1]^。异常的肿瘤血管改变了肿瘤代谢微环境，主要表现为缺氧和酸中毒，进而造成了肿瘤对化疗、放疗以及免疫治疗的抵抗。Jain^[2]^提出了肿瘤血管正常化理论：合理地运用抗血管生成药物，在特定时间窗内使肿瘤血管在结构和功能上趋于正常，最终提高抗瘤效果并抑制肿瘤转移。

肿瘤具有免疫抗原性，免疫系统能识别并杀伤肿瘤细胞。肿瘤免疫经历三个关键的阶段，即“清除”、“平衡”、“逃逸”。在“清除期”，免疫系统监测、抑制肿瘤细胞；随着肿瘤抗原性减弱，免疫系统识别和清除受到限制，但肿瘤又处在免疫系统的清除压力下，以至不能过度生长，二者互相抗衡，进入“平衡期”；肿瘤细胞与免疫系统相互作用，肿瘤基因发生变化、免疫微环境改变，累积达到一定程度，这种平衡就被打破，肿瘤逃逸免疫反应^[3, 4]^。肿瘤免疫治疗就是基于肿瘤的免疫原性，通过主动免疫或被动免疫，达到抑制、清除肿瘤的目的。然而，异常的肿瘤血管却促进免疫抑制微环境的形成，促进肿瘤免疫逃逸的发生。

综上，在促血管生成占优势的肿瘤组织中，新生血管结构及功能的异常，促进了肿瘤免疫抑制微环境的形成。适当的抑制血管生成，正常化肿瘤血管，可以改善免疫抑制的微环境，从而增强肿瘤免疫清除，现就针对血管正常化与肿瘤免疫联合的机制及疗效做如下综述。

## 1 血管形成和肿瘤免疫

### 1.1 异常血管和免疫抑制微环境的形成

血管生成是肿瘤发生发展中的重要事件。在肿瘤组织中，多种转录因子，如缺氧诱导因子（hypoxia inducible factor, HIF）等，可引发促血管生成因子如血管上皮生长因子（vascular endothelial growth factor, VEGF）和血小板源性衍生生长因子的表达。其中VEGF促血管活性最强。已证实它的表达也受到上皮生长因子，*K*-*ras*等癌基因蛋白的调控^[5]^。因此，在癌基因蛋白、缺氧等内外源性因素影响下，促血管生成占优势，血管上皮细胞增生、迁移、新生血管形成。

这些新生的肿瘤血管结构异常，包括僵硬、扭曲、扩张以及PVC的结构异常或缺如，致使血流灌注不足及血管通透性增加，最终形成了高渗、低氧、酸性、高间质压的微环境。这很可能影响免疫细胞的增生、浸润、存活及功能^[2, 6, 7]^。目前的临床前及临床研究中证实，在上述微环境中，促进抗瘤免疫的细胞，如成熟树突状细胞（dendritic cells, DC）相对缺乏，而抑制抗瘤免疫的细胞却相当丰富，如肿瘤相关巨噬细胞（tumor associated macrophage, TAM）、调节T细胞（regulatery T cell, Treg）、髓源抑制性细胞（myeloid-derived suppressor cells, MDSCs）^[4, 8, 9]^。

#### 1.1.1 异常血管抑制树突状细胞（DC）的成熟

VEGF在肿瘤异常血管形成中起着重要作用。VEGF家族有6个不同的成员（VEGF-A、VEGF-B、VEGF-C、VEGF-D、VEGF-E和PIGF）。其中，VEGF-A的作用尤为重要。肿瘤源性VEGF-A通过VEGFR1通路抑制转录因子NF-κB，从而阻止DC成熟^[10, 11]^。Yang等^[12]^对比在缺氧及正常氧供下的DC细胞，证实低氧抑制DC细胞的成熟。成熟DC在抗瘤免疫中发挥重要作用，不成熟的DC尽管仍可提呈包括肿瘤在内的抗原，但不能有效激活T细胞。缺氧抑制DC产生T辅助细胞-1（Th-1），而倾向于生成T辅助细胞-2（Th-2）。Th-1分泌细胞因子IFN-γ，可促进肿瘤特异性细胞毒性T淋巴细胞（cytotoxic T lymphocyte, CTL）的抗瘤作用，是保护性效应细胞。与之相反，Th-2生成IL-4、IL-5和IL-13而抑制CTL对肿瘤抗原的杀伤。可见，无论是VEGF还是异常血管造成的缺氧都不利于DC细胞的成熟。

#### 1.1.2 肿瘤异常血管促进MDSCs聚集

肿瘤组织中，促血管VEGF-A依赖VEGFR2信号通路引起MDSCs聚集及效应T细胞减少^[13]^。血管异常引起组织缺氧，通过HIF-1α上调精氨酸酶（Arginase, Arg）和NO，促进MDSCs向TAMs（M2）转化^[14]^。

肿瘤组织中的MDSCs可直接促进血管生成并有效抑制T细胞介导的抗瘤反应^[15, 16]^。研究表明，MDSCs在多种肿瘤（如乳腺癌、肝癌、成纤维细胞瘤、慢性粒细胞白血病等）中发挥免疫抑制的作用。Yu等^[17]^发现乳腺癌患者的肿瘤组织及外周血中，MDSCs抑制抗瘤免疫反应。同样地，在肝细胞癌患者中也发现MDSCs增加，且清除MDSCs可重塑CD8^+^ T细胞及CD4^+^ T细胞功能^[18]^。

#### 1.1.3 异常血管促进TAMs的极化及Treg浸润

TAMs有至少两种亚型：M1和M2。M1可以通过介导Th-1分泌细胞因子（如IL-12、IFN-γ）激活CTL杀伤肿瘤细胞。相反的，M2表达高水平的Arg-1、IL-10、VEGF和转化生长因子-β，抑制Th-1作用^[9, 19]^。肿瘤组织表面表达的细胞因子促进单核细胞浸润并分化成为TAMs，而异常肿瘤血管直接造成的缺氧，则促进TAMs极化为免疫抑制亚型M2。因此，尽管各种类型的免疫细胞都可以浸润肿瘤实质，而在肿瘤组织中，抑制免疫细胞群更具优势^[20]^。此外，免疫抑制细胞群Treg细胞，通过缺氧介导趋化因子（如CCL-22和CCL-28）^[21]^的上调而优先浸润肿瘤组织。

综上，肿瘤异常血管的生成，促进了免疫抑制微环境的形成。使得肿瘤细胞逃逸免疫系统的清除。

### 1.2 血管正常化改善肿瘤免疫抑制微环境

如前所述，异常血管抑制免疫功能，那么正常化肿瘤血管可否逆转这一过程呢？

#### 1.2.1 基因介导的血管正常化改善免疫抑制微环境

近来，动物研究结果发现，肿瘤血管相关基因改变可以介导血管正常化，增强免疫细胞的浸润及功能，从而改善免疫抑制微环境。一项关于鼠胰岛瘤的模型的研究发现，G蛋白信号调节因子5（regulator of G protein signaling 5, RGS5）的缺乏促进肿瘤血管正常化，增加PVC覆盖及血流灌注，使效应T细胞传递至肿瘤组织，最终延长鼠的生存期^[22]^。另一项研究^[23]^表明，过表达富组氨酸糖蛋白（histidine-rich glycoprotein, HRG）通过PIGF依赖机制增加PCV覆盖率，正常化肿瘤血管，提高血流灌注并改善缺氧。高表达HRG还增加肿瘤组织DC和CD8^+^ T细胞浸润，使得TAMs从M2转化成M1。

#### 1.2.2 药物介导的血管正常化改善免疫抑制微环境

合理运用抗血管药物，促进血管正常化，增强DCs的数量及功能、减少肿瘤组织及循环中的Treg、减少MDSCs的比例，增加CD4^+^T细胞和CD8^+^T细胞的瘤内浸润，最终改善肿瘤免疫抑制微环境^[24-27]^。抗血管药物主要分为两种：酪氨酸激酶抑制剂（TKI），靶向促血管生成相关分子受体以阻碍其信号通路；另一个则是单克隆抗体，直接靶向循环中VEGF或VEGFR。前者的代表性药物舒尼替尼（sunitinib）和索拉非尼（sorafenib），已批准用于结直肠癌、乳腺癌、非小细胞肺癌等多种肿瘤^[28]^。后者的代表性药物贝伐珠单抗（bevacizumab），联合免疫治疗改善包括转移性直肠癌在内的不同类型肿瘤患者的生存期^[29]^。

## 2 血管正常化与免疫治疗的联合运用

成功的免疫治疗不仅需要免疫细胞的浸润，还需要免疫支持的微环境来维持T细胞的增生及功能。血管正常化恰好为免疫治疗提供了这样的环境。因此，血管正常化联合免疫治疗受到越来越多的关注^[30, 31]^。

目前，关于二者联合治疗的研究在多种肿瘤模型中进行，Rajeev等^[26]^在小鼠黑素瘤模型中证实了低剂量抗-VEGF抗体增强获得性细胞免疫治疗的抗瘤作用。另一项研究则通过在MCaP0008乳腺癌模型中使用低剂量的抗-VEGFR2抗体DC101提高肿瘤疫苗治疗疗效，表明血管正常化对免疫治疗的增强作用^[27]^。该促进作用在鼠胰腺神经内分泌瘤模型中也得到证实^[32]^。相似的，在肺癌的鼠模型中，重组人内皮抑素正常化肿瘤血管联合细胞因子诱导的杀伤细胞可以抑制肺癌的生长^[33]^。

然而，要成功将二者的联合治疗运用于临床还面临着挑战。首先，血管正常化的时间窗较难把握。因此，寻找可靠的标志物，有助于在最佳血管正常化时间窗内联合免疫治疗。研究表明，肿瘤组织CD8^+^ T细胞浸润和血管正常化有很强的相关性，有望成为血管正常化的有效生物标志物^[27]^。其次，血管正常化剂量的选择也存在困难，过度的抗血管治疗同样抑制抗瘤免疫，目前倾向小剂量运用抗血管药物^[26, 27]^，但具体剂量的制定还有待进一步研究。此外，肿瘤是高度异质的，在不同的肿瘤以及肿瘤的不同分期，需要靶向不同的促血管生成因子，并且针对不同的患者及疾病状态选择的剂量也不尽相同，因此更加注重个体化治疗，这也给临床实际应用带来了困难。

## 3 展望

肿瘤血管的异常导致低氧、酸性、高间质压微环境的形成，该微环境促进肿瘤生长、转移，并通过介导免疫抑制细胞TAMs、MDSCs等的产生限制了免疫治疗的疗效。研究表明，合理运用抗血管药物，正常化肿瘤血管可以使该微环境向免疫支持转化，促进效应T细胞浸润并发挥作用，从而增强免疫治疗，尤其是获得性免疫治疗的疗效。

目前二者联合治疗的研究多在动物模型中进行，未来将进行更多的临床研究。此外，生物标志物的研究将成为一个重要的方向，它有助于发现治疗优势人群及最佳用药时机，相信经过不断深入的研究，抗血管药物联合免疫治疗有望为抗瘤治疗开辟新方向。
